# Dietary isobutyric acid supplementation improves intestinal mucosal barrier function and meat quality by regulating cecal microbiota and serum metabolites in weaned piglets

**DOI:** 10.3389/fvets.2025.1565216

**Published:** 2025-05-02

**Authors:** Binjie Wang, Junjie Hou, Yundong Cao, Haibo Wei, Kangle Sun, Xiang Ji, Xiaoran Chu, Yu Zhang, Sen Jiang, Linlin Shi, Ke Liu, Zhen Song, Fengyun Wen

**Affiliations:** ^1^College of Animal Science and Technology, Henan University of Science and Technology, Luoyang, Henan, China; ^2^Anqiu County Animal Husbandry Development Center, Weifang, Shandong, China; ^3^The Kay Laboratory of High Quality Livestock and Poultry Germplasm Resources and Genetic Breeding of Luoyang, Luoyang, Henan, China

**Keywords:** isobutyric acid, intestinal mucosal barrier function, gut microbiota, intramuscular fat, serum metabolome, weaned piglets

## Abstract

This study aimed to provide evidence for the effects of isobutyric acid on the intestinal mucosal barrier and gut microbiota in weaned piglets. In this study, 30 piglets were divided into two groups: one group was fed a standard diet (CON group), and the other group was fed a diet supplemented with 0.5% isobutyric acid (IB group) for 21 days. The results showed that isobutyric acid significantly increased (*p* < 0.05) serum immunity and antioxidant capacity in weaned piglets. In small intestine of piglets, the ratio of villus height to crypt depth was significantly increased (*p* < 0.05). Administration of isobutyric acid also increased (*p* < 0.05) the expression of genes related to intestinal mucosal barrier function. Cecal microbiota analysis revealed that isobutyric acid significantly increased (*p* < 0.05) the abundance of the *Eubacterium coprostanoligenes* group. Untargeted serum metabolomics analysis indicated that the top three categories of metabolites were lipids and lipid-like molecules, organic acids and derivatives, and organic heterocyclic compounds. Additionally, in longissimus thoracis muscle, isobutyric acid significantly increased (*p* < 0 0.05) intramuscular fat and triglyceride content compared with the CON group. Overall, isobutyric acid can improve small intestinal mucosal barrier function, and may influence the fat deposition through the regulation of serum metabolites in weaned piglets.

## 1 Introduction

Weaning is a challenge for piglets, which are prone to intestinal stress due to their underdeveloped digestive system and changes in diet and environment (1). Damage to the intestinal barrier can affect paracellular permeability, disrupt tight junctions, and increase the risk of bacterial translocation and intestinal infection, subsequently leading to intestinal stress (2). During the weaning phase, the proteins in solid feeds neutralize the piglets’ gastric acids, leading to an increase in the pH of the piglets’ digestive tract and a decrease in the activity of digestive enzymes (3). During this period, piglets take less food, which subsequently leads to less nutrients being digested and absorbed and an increased risk of diarrhea (4, 5). Over the past few decades, the farming industry has addressed this problem by adding antibiotics to feed rations (6). With the banning of antibiotics in the livestock industry, a number of feed additives with beneficial effects were gradually used in the pig industry.

Meats are rich in proteins, amino acids, vitamins and trace elements needed by the human body to provide energy. Pork is the most widely consumed meat globally, and holds a significant place in human dietary culture (7). In recent years, there has been a growing demand for pork quality (8). In contrast, high-quality meats are more favored by consumers. The muscle with higher intramuscular fat content often exhibits lower shear force and higher scoring marble pattern, representing higher meat quality. Intramuscular fat is defined as the fat located between muscle fiber bundles. Physiologically, intramuscular fat is considered to have similar functions as other fat depots in the body, such as energy storage (9). As a food, intramuscular fat greatly enhances the texture and flavor of pork, satisfying consumer demands while also attracting more purchases. The intramuscular fat content is a key indicator for evaluating the quality of pork. The urgent problem that needs to be solved in the pig farming industry is how to increase the intramuscular fat content of pork and improve its quality.

Gut microbiota is an important environmental factor that affects energy harvest from the diet and energy storage in the host. Studies of GF and conventionalized mice revealed that the microbiota promotes absorption of monosaccharides from the gut lumen, with resulting induction of *de novo* hepatic lipogenesis (10). Metagenomic sequencing revealed that high-fat pigs had a higher abundance of Archaeal species with methanogenesis functions, leading to more-efficient fat deposition, while low-fat pigs had higher abundances of butyrate-producing bacteria species that improved the formation of SCFAs, especially butyrate, thus alleviating fat deposition in pigs (11). Increased oxidative stress associated with fat consumption may alter the bacterial composition and the expression of lipogenic genes. GSH-Px levels were significantly lower in high-fat pigs, which are more susceptible to oxidative stress than low-fat pigs, and hence more susceptible to obesity (12).

Short-chain fatty acids (SCFA) are important metabolic regulators as energy-providing substrates. The composition of dietary fatty acids affects the digestion, absorption and metabolism of nutrients (13). Branched chain fatty acids (BCFA) are primarily saturated fatty acids with one or more methyl branches on the carbon chain, which are components of common food fats (14). BCFA reduced the incidence of necrotizing enterocolitis and increased the intestinal anti-inflammatory cytokine IL-10, and altered gastrointestinal microbial ecology in a neonatal rat model (15). Isobutyric acid is one of the microbial breakdown products of indigestible carbohydrates (16). Exogenous supplementation with butyric acid or butyrate improves intestinal barrier function and promotes intestinal health in weaned piglets (17). Addition of fatty acid blend products to maternal rations improves offspring performance (18). Several studies have shown that short-chain fatty acid levels increase in obese individuals and decrease after weight loss (19). This suggests that increased levels of short-chain fatty acids increase the amount of energy obtained from the diet.

Straight-chain fatty acid modulate the composition of gut microbiota, and are thought to affect the intestinal health; however, very little is known about BCFA influence on intestinal mucosal barrier function and intramuscular fat deposition in weaned piglets. Although isobutyric acid has been reported as a dietary additive for ruminants (20, 21), its application in weaned piglets remains limited, and its effects on the intestinal mucosal barrier and gut microbiota of weaned piglets are not yet well understood. Therefore, this study aims to investigate the impact of dietary supplementation with isobutyric acid on the intestinal mucosal barrier function of weaned piglets.

## 2 Materials and methods

### 2.1 Ethics approval

The animal study was approved by the Institutional Animal Ethics Committee of Henan University of Science and Technology (approval 2022-02-023).

### 2.2 Animals and experimental design

All procedures involving the use of animals were performed in accordance with the animal protection and welfare guidelines established by the Chinese Ministry of Agriculture. The rearing trial was conducted at Henan University of Science and Technology experimental animal farm in Luoyang city, Henan province. In this study, 30 weaned pigs (Large White pigs) with an average body weight of 10 ± 2 kg were reared at the unified farm of Henan University of Science and Technology in Luoyang City, Henan Province, and fed with suckling pig compound feed twice a day, with free drinking water. After 10 days of acclimatization to the environment, the pigs were divided into two treatment groups: a control group (CON group, fed with basal diet) and an isobutyric acid group (IB group, fed a diet with 0.5% isobutyric acid added to the basal diet). Supplementary Table S2 shows the composition and nutrients of the basal diet performed by National Research Council nutrient requirements (61). Isobutyric acid (Macklin I914990, Shanghai, China) was purchased from the Shanghai Macklin Biochemical Technology Company. The trial period was 21 days. At the end of the experiment, three pigs were randomly selected from each group. The pigs were fasted for 24 h and euthanized with an anesthetic injection given into the ear vein with an overdose of Alfaxan (0.7 mg/kg), and blood samples were collected. After slaughter, approximately 200 g of longissimus thoracis (LT) and backfat tissue at the fifth to sixth rib was promptly collected, immediately preserved in liquid nitrogen, and then stored in a refrigerator set at −80°C. Each intestinal segments (duodenum, jejunum, ileum, and cecum) were also isolated and collected in a refrigerator at −80°C.

### 2.3 Analysis of serum biochemistry, immunoglobulin, and antioxidative index

The activity of total antioxidant capacity (T-AOC) and glutathione S-transferase (GST), and superoxide dismutase (SOD) and malondialdehyde (MDA) concentration in serum was measured using biochemical methods following manufacturer’s instructions provided for each reagent kit (Beijing Solarbio Science & Technology Co., Ltd., Beijing, China). Briefly, the content of MDA was determined by the thiobarbituric acid reaction. The activity of T-AOC, GST, and SOD were assayed using the Fe^3+^-Tripyridyltriazine, the dithiodinitrobenzoic acid colorimetric, and xanthine oxidase, respectively. The concentrations of leptin (LEP), cholecystokinin (CCK), growth hormone (GH), glucocorticoid receptor (GR), Ghrelin, adiponectin (ADP), and insulin (INS) in serum were determined by the commercial ELISA kit (Wuhan ColorfulGene Biological Technology Co., LTD., Wuhan, China). And the concentrations of immunoglobulin A (Ig A), immunoglobulin G (Ig G), and immunoglobulin M (Ig M) in serum were determined by the commercial ELISA kit (Wuhan ColorfulGene Biological Technology Co., LTD.). All procedures were performed according to the manufacturer’s instructions.

### 2.4 Histological analysis

For morphology, the abdomen was opened after slaughter, and the duodenum, jejunum, and ileum were removed and rinsed with phosphate buffer solution (PBS), and the intestinal segments were preserved in 4% paraformaldehyde solution before embedding, and consecutive segments of 5 μm thickness were counterstained with hematoxylin and eosin (H&E) (22).

Morphological changes were observed under a DM300 light microscope from Leica (Germany). For analysis, 10 crypts were randomly selected from different parts of the section, and their crypt depths and corresponding villus heights, the number of goblet cells were measured and their ratios were calculated using ImageJ v1.8.0 software.

### 2.5 Determination of meat quality

After slaughter, backfat thickness, marbling score, pH, meat color, IMF content, and the triglyceride (TG) content of muscle and backfat were measured. The average backfat thickness of the first rib, last lumbar spine, and last rib were used to define the backfat thickness. The marbling score was determined from a cross-sectional slice of the muscle at the first lumbar spine and ranged from 0 to 5, with higher scores indicating more abundant IMF content. The color of LT was determined using OPTO-STAR (Germany, Denmark), and the meat color parameters included L* (lightness), a* (intensity), and b* (yellowness).

The pH value of LT was determined 40–50 min after slaughter (HI99163, HANNA, Italy). The IMF content was measured using a Soxhlete extractor (SX-360, OPSIS, Sweden). Triglyceride content was determined using a triglyceride kit (Beijing Solarbio Science & Technology Co., Ltd., Beijing, China), following manufacturer’s instructions.

### 2.6 Analysis of the microbial composition and diversity of the cecal contents

Bacterial DNA from cecal contents was extracted using Fecal DNA Extraction Kit Solarbio D2700-50 (Solarbio Bio-Tek, Beijing, China) according to manufacturer instructions. Extracted DNA was assessed using 1% agarose gel electrophoresis and Nanodrop-2000 (Thermo Scientific). The barcode primers were 341 F(5’-CCTACGGGNGGCWGCAG-3′), and 805 5′-R(5GACTACHVGGGTATCTAATCC-3′). These primers were used to amplify the V3–V4 hypervariable region of the 16S rRNA. The PCR products were quantified using the QuantiFluor™ -ST Blue fluorescent Quantitative System (Promega), followed by Illumina PE250 library construction and Illumina PE250 sequencing. The PE reads obtained by Illumina PE250 sequencing were first assembled according to the overlap relationship, and sequence quality was controlled and filtered simultaneously. After distinguishing the samples, OTU cluster and taxonomic analyses were performed. The sequencing depth was determined based on taxonomic information, and statistical analyses of the community structure were performed at each taxonomic level (23). The sequence data can be found in National Center for Biotechnology Information (accession number PRJNA1246593). The correlation analysis was conducted between gut microbiota and intestinal morphology, IMF, TG.

### 2.7 Untargeted metabolomics assays

Relevant experimental methods and procedures for metabolomics performed upon serum can be found in previous studies (24, 25). Briefly, 200 mg serum samples were mixed with 2-chlorophenylalanine (4 ppm) methanol (−20°C) and ground by a high-throughput tissue grinder for 90 s at 60 Hz. The samples were centrifuged at 4°C for 10 min at 12,000 rpm, and the supernatant was filtered through 0.22 μm membrane to obtain the prepared samples for LC–MS. Twenty microlitre from each sample were taken to the quality control samples, and the rest of the samples were used for LC–MS detection.

The pre-processed data were converted to the Statistics Analysis files and then processed using SIMCA software (V16.0.2, Sartorius Stedim Data Analytics AB, Umea, Sweden). Orthogonal projections to latent structures- discriminant analysis (OPLS-DA) was conducted as a supervised method to identify the significant variables with discriminatory capability, and validity of the OPLS-DA model was corroborated by a permutation test with a 200-time repetition. The variable importance in the projection (VIP) value of each variable from the OPLS-DA model was computed to demonstrate its contribution to the classification. Metabolites with the VIP value>1 were further analyzed in Student’s *t*-test at univariate level to evaluate the significance of each metabolite. Metabolites with *p* value<0.05 and fold change >1.5 were regarded to have statistical significance. To assess the differentially expressed metabolites, the volcano plot was made for the two groups. What’s more, Pearson correlation coefficient was calculated to analyze the correlation between all metabolites and the results were shown in the correlation heatmap. Furthermore, the Kyoto Encyclopedia of Genes and Genomes (KEGG) annotation analysis as well as the pathway analysis (including enrichment analysis and network analysis) was conducted for identification of the differentially expressed metabolites. The sequence data can be found in China National Center for Bioinformation (accession number PRJCA038200). The correlation analysis was conducted serum metabolism and IMF, TG.

### 2.8 Total RNA extraction and qPCR

TRIzol reagent (Servicebio, China) was used to extract total RNA from intestine and LT, and a spectrophotometer (NanoDrop 2000, Thermo Fisher) was used to determine the absorbance of RNA at 260 nm and 280 nm to ensure the purity and concentration of RNA. The RNA was then reverse-transcribed using the SweScript RT I First Strand cDNA Synthesis Kit (Servicebio, China) and random primers. QPCR was performed using a Bio-Rad CFX Connect PCR fluorescence quantitative PCR instrument with SYBR Green qPCR Master Mix and gene-specific primers (Supplementary Table S1), and relative expression was calculated using the 2^−ΔΔCt^ method (26).

### 2.9 Western blot analysis

The protein expression levels of longissimus thoracis FASN, ACC, LPL, HSL, FABP4, *β*-actin were determined. The total proteins were isolated using the RIPA buffer with PMSF and a phosphatase inhibitor. The protein concentration was quantified using the bicinchoninic acid (BCA) protein assay kit (Servicebio, China). Protein separation was conducted by 8% (12%) sodium dodecyl sulfate-polyacrylamidegel electrophoresis (SDS-PAGE). The separated proteins were transferred to the polyvinylidene fluoride (PVDF) membrane by a wet tank transfer blotting system (Bio-Rad Laboratories). The PVDF membrane containing proteins was cut with a blade according to the location of the target protein and blocked using 5% skimmed milk for 30 min at room temperature. Then, the membrane was incubated with specific primary antibodies overnight at 4°C, followed by secondary antibodies for 2 h at room temperature. The blots were quantified using the Gel imaging system software (Image Lab 5.2, Bio-Rad).

### 2.10 Statistical analysis

Statistical analyses were performed using SPSS 22.0. GraphPad software (9.0), and Origin 2022 were used for correlation analysis and data visualization, and *p* values less than 0.05 were considered significant. Growth performance was analyzed by one-way ANOVA, and other experimental results were compared by *t*-test. Data are represented in tables and graphs as mean and standard deviation (SD) (*n* = 3).

## 3 Results

### 3.1 Effects of isobutyric acid supplementation on serum physiochemical parameters, immunoglobulin level, and antioxidative index in weaned piglets

**Figure 1:**
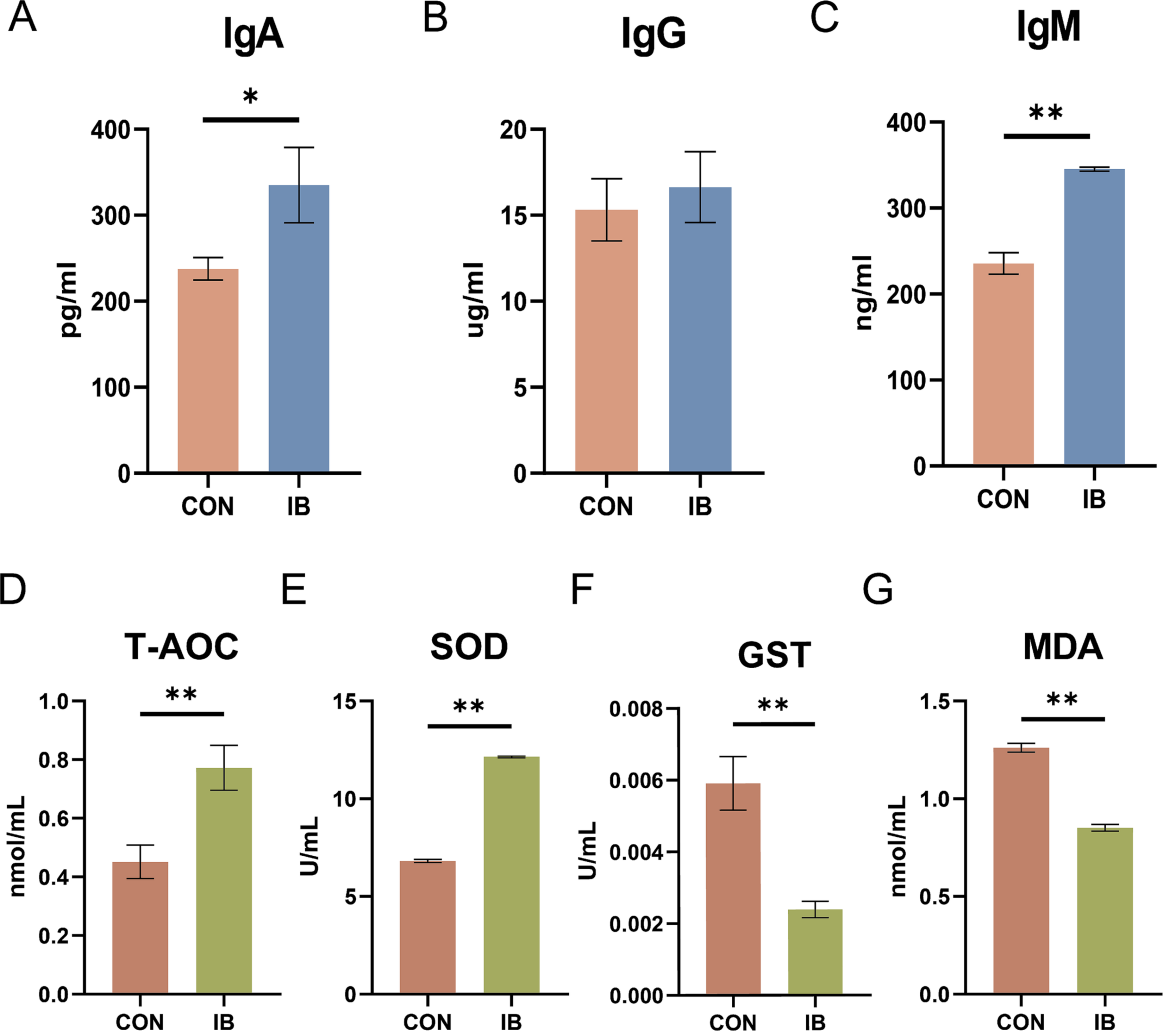
Effect of isobutyric acid on immunoglobulin level and antioxidant capacity of weaned piglets. **(A)** Immunoglobulin A (Ig A); **(B)** Immunoglobulin G (Ig G); **(C)** Immunoglobulin M (Ig M); **(D)** Total antioxidant capacity (T-AOC); **(E)** Superoxide dismutase (SOD); **(F)** Glutathione S-transferase (GST); **(G)** Malondialdehyde (MDA).* *p* < 0.05 and ** *p* < 0.01.

The effects of dietary isobutyric acid on serum physiochemical parameters in weaned piglets are present in Table 1. Isobutyric acid administration caused a decrease in serum ADP (*p* < 0.01). Compared to the CON group, a diet with 0.5% isobutyric acid supplementation increased serum immunoglobulin A (Ig A) (*p* < 0.05) and immunoglobulin G (Ig G) (*p* < 0.05) levels (Figure 1).

**Table 1:** Effect of isobutyric acid on serum physiochemical parameters of weaned piglets.

Items	CON^1^	IB^1^	SEM^2^	*p*-value
LEP, pg.·mL^−1^	446.6 ± 49.81	521.7 ± 25.67	22.17	0.08
CCK, pg.·mL^−1^	220.1 ± 21.65	232.1 ± 29.88	9.89	0.6
GH, pg.·mL^−1^	2,879 ± 479.1	2,648 ± 140.2	138.8	0.47
GR, pg.·mL^−1^	1,088 ± 90.75	1,113 ± 82.26	32.13	0.74
Ghrelin, pg.·mL^−1^	2,363 ± 164.4	2,109 ± 37.02	71.55	0.059
ADP, ng·mL^−1^	27.02 ± 0.41	19.90 ± 1.18	1.625	0.0006
INS, mU·L^−1^	30.49 ± 1.19	29.53 ± 2.32	0.71	0.56

1 CON, control group; IB, isobutyric acid group.

2 SEM, standard error of the mean.

LEP, leptin; CCK, cholecystokinin; GH, growth hormone; GR, glucocorticoid receptor; ADP, adiponectin; and INS, insulin. Results are presented as mean ± SD, *n* = 3. * *p* < 0.05 and ** *p* < 0.01.

As presented in Figure 1, piglets fed diets containing 0.5% isobutyric acid had higher total antioxidant capacity (T-AOC) (*p* < 0.05) level. Moreover, isobutyric acid treatments exhibited a decrease in serum glutathione S-transferase (GST) activity (*p* < 0.05) but an increase in serum superoxide dismutase (SOD) activity (*p* < 0.01). We also investigated the serum malondialdehyde (MDA) level. As expected, a diet supplemented with 0.5% isobutyric acid significantly reduced the serum MDA concentration (*p* < 0.01). Our findings indicated that isobutyric acid could enhance immunity and antioxidant capacity in weaned piglets.

### 3.2 Effect of isobutyric acid on small intestinal mucosal barrier function in weaned piglets

**Figure 2:**
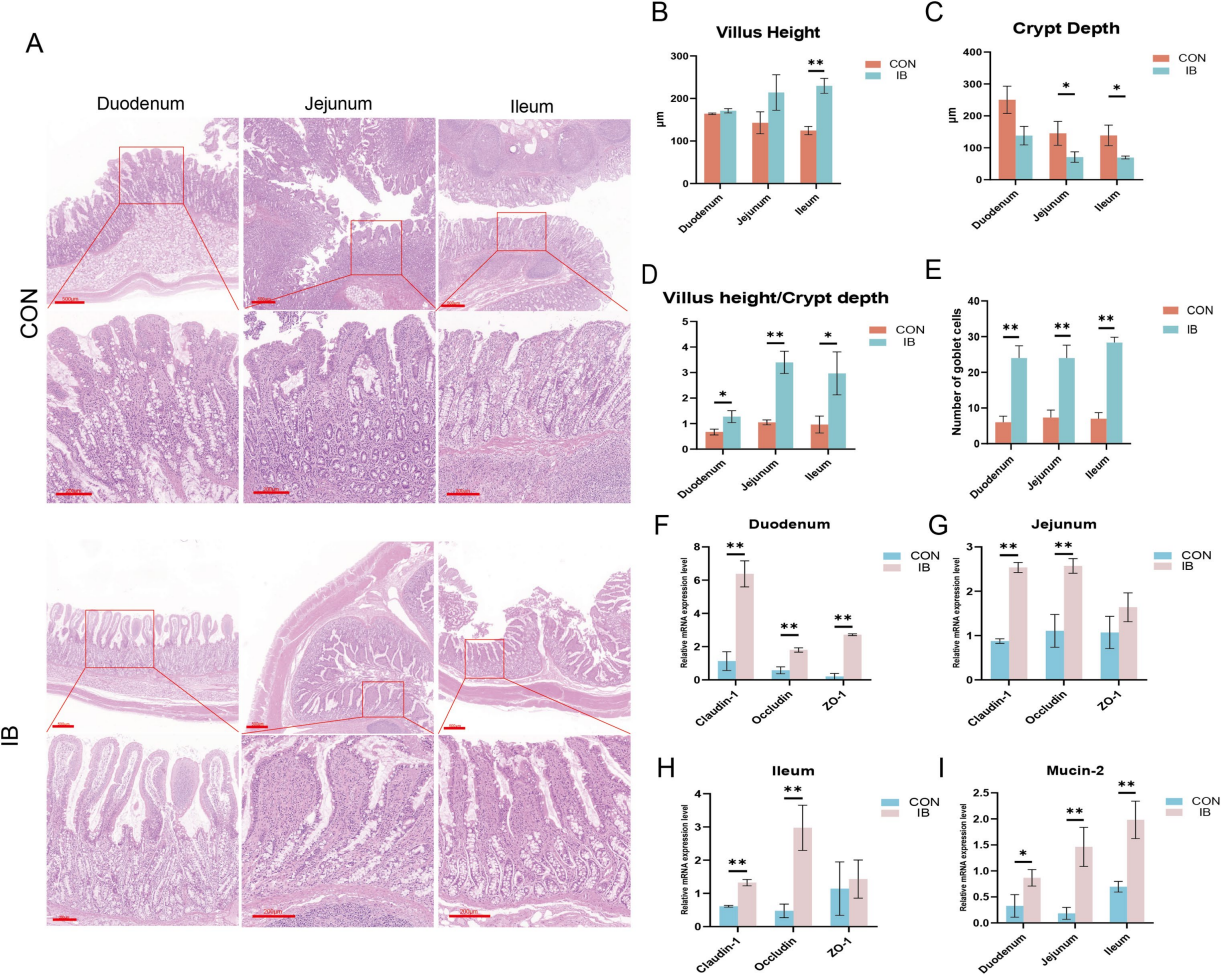
Effect of isobutyric acid on small intestinal mucosal barrier function. **(A)** HE staining shows the morphology of various segments of the intestine; **(B)** Villus height of the duodenum, jejunum and ileum; **(C)** Crypt depth of the duodenum, jejunum and ileum; **(D)** Ratio of villus height to crypt depth of the duodenum, jejunum and ileum; **(E)** Number of goblet cells of the duodenum, jejunum and ileum; **(F)** The relative mRNA expression levels of *Claudin-1*, *Occludin* and *ZO-1* in duodenum between the CON and IB groups; **(G)** Jejunum; **(H)** Ileum; **(I)** The relative mRNA expression levels of *MUC2* in duodenum, jejunum and ileum. * *p* < 0.05 and ** *p* < 0.01.

Representative images of HE staining are shown as the picture (Figure 2A). We found that isobutyric acid increased villus height (VH) and decreased crypt depth (CD) in duodenum (Figures 2B,C). Compared with CON group, we observed isobutyric acid significantly reduced CD (*p* < 0.05) in jejunum and ileum (Figure 2C). Interestingly, the HE staining only showed a significantly increasing of VH (*p* < 0.05) in ileum (Figure 2B). In addition, we calculated the ratio of villus height to crypt depth (VCR) in each part of the small intestine. The results showed that the VCR was significantly increased in all parts of the small intestine (*p* < 0.05, *p* < 0.01) compared to the CON group (Figure 2D). We also calculated the number of goblet cells (GCs). And we found the GCs in each intestinal segment was significantly higher than CON group (*p* < 0.05) (Figure 2E).

The significant differences in small intestinal morphology between the two groups made us wanna explore the effects of isobutyric acid on the mucosal barrier of the small intestine. To demonstrate whether dietary supplementation with isobutyric acid positively affects the small intestinal barrier, we measured the genes related to tight junction protein, including *Claudin-1*, *Occludin* and Zonula occludens-1 (*ZO-1*). Compared with the CON group, the expression of *Claudin-1*, *Occludin* and *ZO-1* were significantly upregulated (*p* < 0.01) in duodenum in the IB group (Figure 2F). The results also showed that the expression of *Claudin-1* and *Occludin* are significantly upregulated (*p* < 0.01) in jejunum (Figure 2G), as well as in ileum (Figure 2H). Meanwhile, we further measured the expression of mucin-2 (*MUC2*). *MUC2* is the major glycoprotein in intestinal mucus. The expression of *MUC2* showed significantly upregulated (*p* < 0.05) in all small intestinal segments (Figure 2I). These findings indicated that dietary isobutyric acid improves small intestinal mucosal barrier function, and promotes intestinal health in piglets.

### 3.3 Effect of isobutyric acid on meat quality and lipid traits in LT of weaned piglets

Compared with the CON group, we confirmed the better meat quality in IB group, as evidenced by the higher marbling score indice (*p* < 0.05). In addition, isobutyric acid significantly increased intramuscular fat (IMF) (*p* < 0.05) and triglyceride (TG) content (*p* < 0.05) in longissimus thoracis (LT) muscle (Table 2). And growth performance are shown in Supplementary Table S3.

**Table 2:** Effects of isobutyric acid on the meat quality of weaned piglets.

Meat quality traits	CON^1^	IB^1^	SEM^2^	*p*-value
Lightness (L*)	49.41 ± 3.56	50.93 ± 5.17	1.66	0.70
Redness (a*)	13.03 ± 0.80	12.99 ± 0.62	0.26	0.95
Yellowness (b*)	6.12 ± 0.06	6.01 ± 0.04	0.03	0.06
Marbling score^3^	3.28 ± 0.14	4.44 ± 0.36	0.28	0.006
pH (45 min)	6.44 ± 0.11	6.39 ± 0.06	0.03	0.58
Shear force, N	50.33 ± 2.48	45.91 ± 1.79	1.27	0.07
IMF, %	1.263 ± 0.36	2.53 ± 0.104	0.29	0.004
BFT, mm	14 ± 3.00	17 ± 5.19	1.68	0.53
Muscle TG, mg/g	0.65 ± 0.08	1.86 ± 0.47	0.29	0.01
Backfat TG, mg/g	94.89 ± 0.55	95.39 ± 1.05	0.32	0.5

1 CON, control group; IB, isobutyric acid group.

2 SEM, standard error of the mean.

3 Marbling score, Marbling score ranges from 1 to 5; higher scores indicate more abundant intramuscular fat.

IMF, intramuscular fat; BFT, backfat thickness; TG, triglyceride. Results are presented as mean ± SD, *n* = 3. * *p* < 0.05 and ** *p* < 0.01.

**Figure 3:**
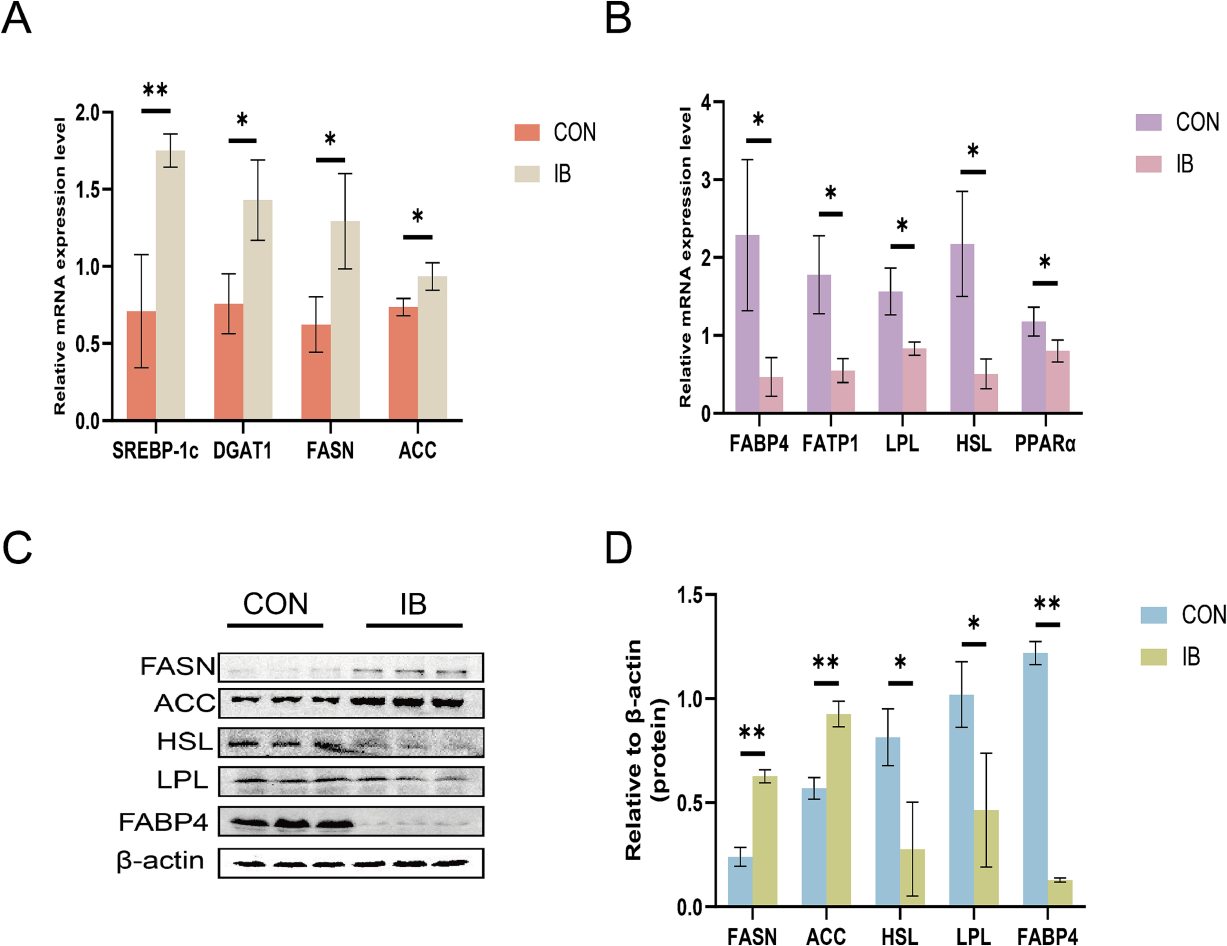
Effect of isobutyric acid on the expression of genes and proteins involved in lipid metabolism. **(A)** Effect of isobutyric acid on the relative mRNA expression of lipid synthesis; **(B)** Effect of isobutyric acid on the relative mRNA expression of lipid breakdown and transport; **(C)** Western blot showing the protein level; **(D)** Bar plots showing the densitometric values. * *p* < 0.05 and ** *p* < 0.01.

Having demonstrated the elevation of lipid traits in LT on weaned piglets, we next sought to examine the expression of genes related to lipid metabolism. As shown in Figure 3, the expression of fatty acid synthase (*FASN*) and acetyl-CoA carboxylase (*ACC*) were significantly upregulated (*p* < 0.05), whereas the expression of peroxisome proliferator-activated receptor *α* (*PPARα*) and hormone-sensitive lipase (*HSL*) were significantly downregulated (*p* < 0.05). And the expression of sterol regulatory element binding protein 1c (*SREBP-1c*) (*p* < 0.01) and diacylglycerol-O-acyltransferase 1 (*DGAT1*) (*p* < 0.05) were significantly upregulated, whereas the expression level of fatty acid transport protein 1 (*FATP1*), fatty acid-binding protein 4 (*FABP4*) and lipoprotein lipase (*LPL*) were significantly downregulated (*p* < 0.05).

### 3.4 Effect of isobutyric acid on gut microbiota in weaned pigs

**Figure 4:**
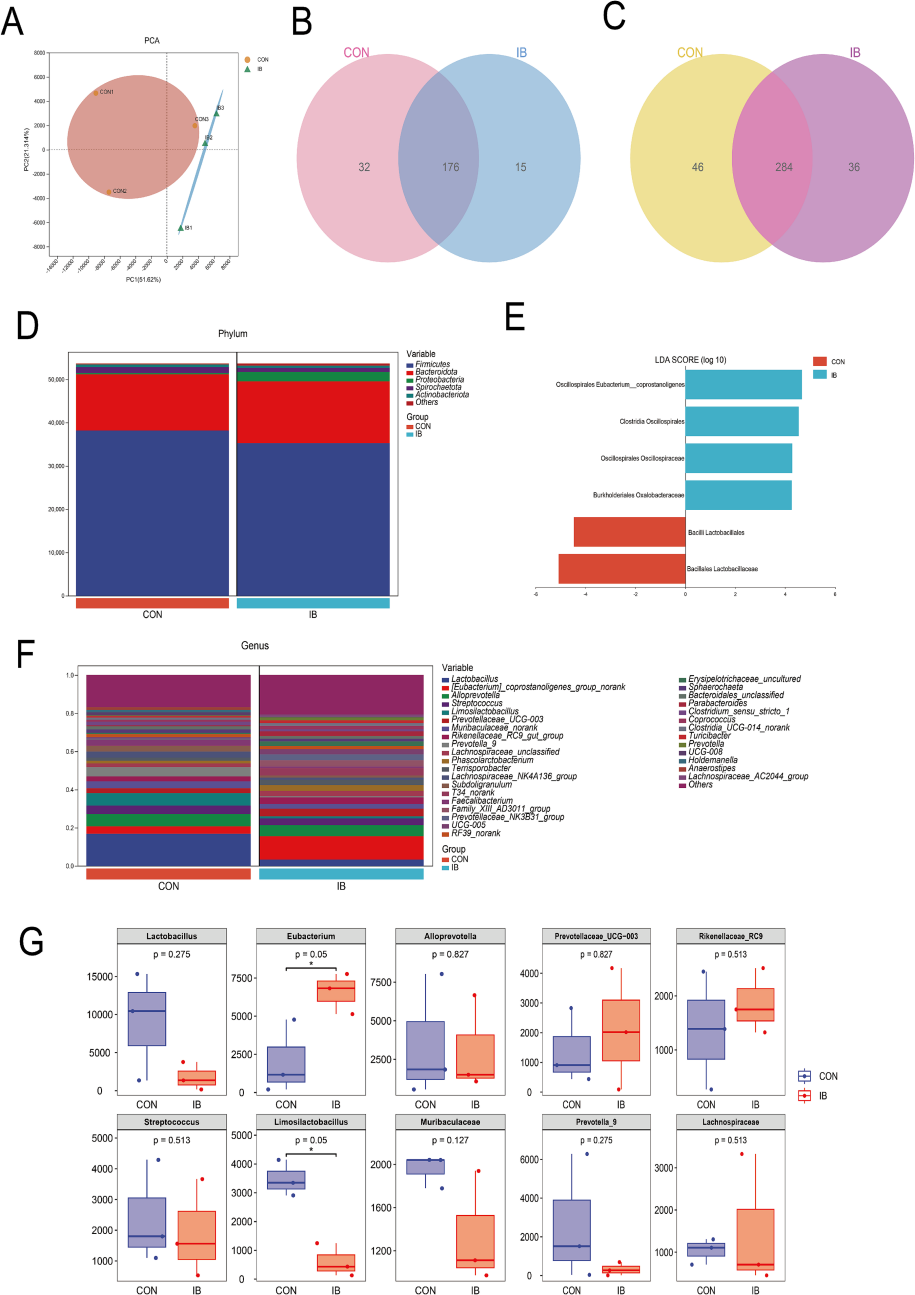
Diversity analysis between CON and IB group. **(A)** Principal component analysis (PCA); **(B)** Venn at the genus level; **(C)** Venn at the species level; **(D)** The proportion of bacteria at the phylum level; **(E)** Linear discriminant analysis Effect Size (LEfSe); **(F)** The proportion of the top 20 bacteria at the genus level; **(G)** The top 10 bacterial abundance at the genus level in boxplot.

We detected and screened 659,837 sequences. OTU analysis of non-repetitive sequences with 97% similarity obtained 817 OTUs, and the rarefaction curve tended to be flat (Supplementary Figures S1A,B). The result indicated that the amount of sequencing for each sample in this study is sufficient. The results of Alpha diversity analysis showed no significant difference in the IB group compared with the CON group, including the Ace index, Chao1 index, Shannon index and Simpson index (Supplementary Figure S1C). Principal component analysis (PCA) revealed clear separation between the microbial communities between the CON and IB groups (Figure 4A). Venn diagrams displayed that 176 genera and 284 species overlapped between the CON and IB groups (Figures 4B,C).

Gut microbiota at the phylum level consisted mostly of Firmicutes, Bacteroidota, Proteobacteria, Spirochaetota and Actinobacteriota. Firmicutes and Bacteroidota represented more than 90% relative abundance (Figure 4D). To further understand the detailed variation in gut microbiota, the relative abundance changes at the genus level were analyzed (Figure 4F). Feeding a diet with isobutyric acid supplementation significantly increased the relative abundance of [Eubacterium]_coprostanoligenes (*p* < 0.05) (Figure 4G). [Eubacterium]_coprostanoligenes belongs to Eubacteriaceae, Firmicutes. Increased levels of Eubacterium may in favor of maintenance of intestinal homeostasis. Limosilactobacillus was significantly decreased (*p* < 0.05) in the IB group. These findings showed that isobutyric acid has a regulatory role in balancing probiotics and pathogenic bacteria in gut microbiota. Furthermore, Linear discriminant analysis Effect Size (LEfSe) was used to determine bacteria with significant differences between the CON and IB groups (Figure 4E). Firmicutes phylum occupied a dominant position in both groups, including three species from the order Oscillospirales and two species from the order Lactobacillales. In general, these changes induced by isobutyric acid help promote intestinal health.

### 3.5 Correlation analysis between gut microbiota and intestinal morphology, IMF, TG

**Figure 5:**
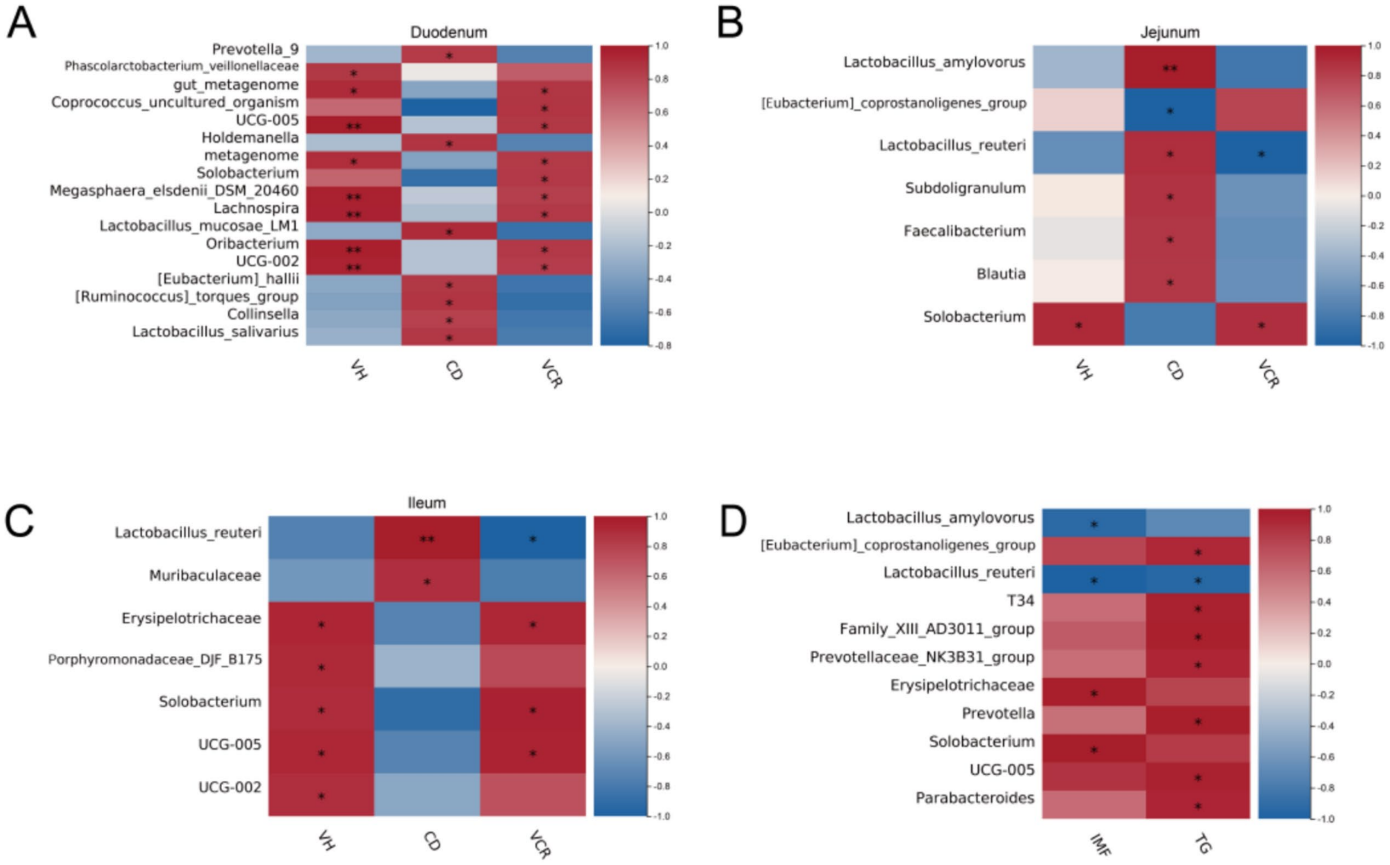
Correlation analysis on cecal microbiota. **(A)** Heat map of the correlation between the differential bacteria and intestinal morphology in duodenum; **(B)** Heat map of the correlation between the differential bacteria and intestinal morphology in jejunum; **(C)** Heat map of the correlation between the differential bacteria and intestinal morphology in ileum; **(D)** Heat map of the correlation between the differential bacteria and lipid traits in LT.

To further investigate the effect of cecal microbial composition on gut morphology, we correlated all microorganisms detected with gut morphology indicators. The results showed that Solobacterium, UCG-002, UCG-005 were significantly and positively correlated with the VCR (*p* < 0.05) of small intestinal segments, as well as Lactobacillus_mucosae_LM1, Lactobacillus_salivarius, Lactobacillus_amylovorus, Lactobacillus_reuteri were significantly positively correlated with CD (*p* < 0.05) (Figures 5A–C). We further explored the relationship between the cecal microbial composition and lipid traits. We found that Solobacterium showed a significant positive correlation with IMF, whereas [Eubacterium]_coprostanoligenes_group, T34, Family_XIII_AD3011_group, Prevotellaceae_NK3B31_group. Prevotella, UCG-005, Parabacteroides showed significant positive correlation with muscle TG content (Figure 5D).

### 3.6 Untargeted metabolomics analysis of serum

**Figure 6:**
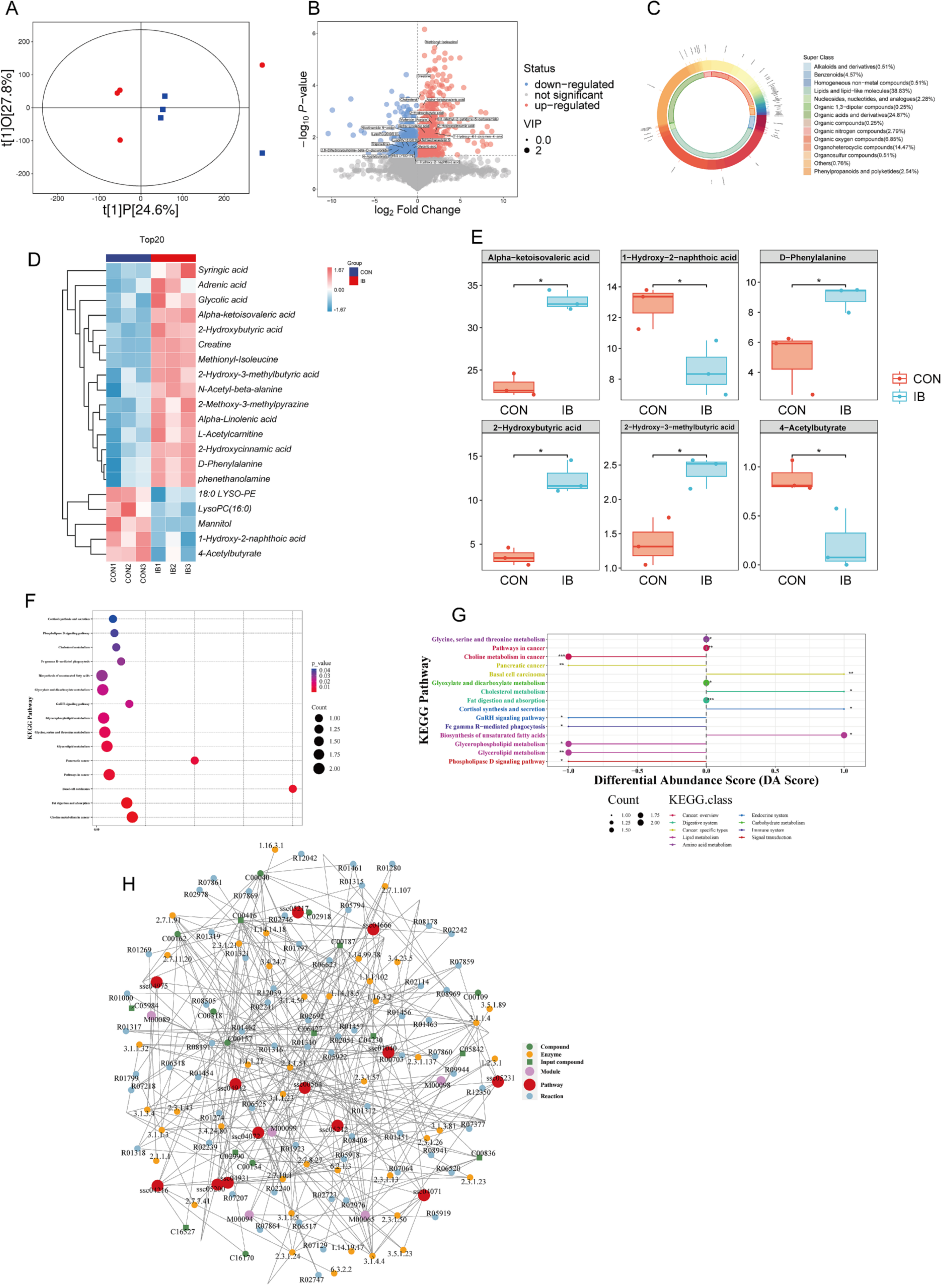
Effect of isobutyric acid on serum metabolites. **(A)** OPLS-DA; **(B)** Volcano plot of serum metabolites; **(C)** Donut plot of metabolite classification and proportion; **(D)** Heatmap of the top 20 serum metabolites; **(E)** The top six serum metabolites in boxplot. **(F)** KEGG enrichment bubble for differential metabolites; **(G)** Differential abundance score for differential metabolites; **(H)** Network analysis for differential metabolites.

Subsequently, serum untargeted metabolomics analysis showed that there were significant differences in serum metabolites between the two groups (Figure 6A), of which 706 metabolites were significantly up-regulated (*p* < 0.05) and 415 metabolites were significantly down-regulated (*p* < 0.05) (Figure 6B). The top three classes with the highest proportion were Lipids and lipid−like molecules, Organic acids and derivatives, and Organoheterocyclic compounds (Figure 6C). In this study, the top 20 serum metabolites were selected for differential analysis, of which 15 were significantly up-regulated (*p* < 0.05) and 5 were significantly down-regulated (*p* < 0.05) (Figure 6D). The six most abundant differential serum metabolites were Alpha-ketoisovaleric acid, 1-Hydroxy-2-naphthoic acid, D-Phenylalanine, and 2-Hydroxybutyric acid, 2-Hydroxy-3-methylbutyric acid, and 4-Acetylbutyrate, among which 1-Hydroxy-2-naphthoic acid and 4-Acetylbutyrate were significantly down-regulated (*p* < 0.05), the remaining four were significantly up-regulated (*p* < 0.05) (Figure 6E). KEGG pathway enrichment analysis showed that Serum differential metabolites were significantly enriched (*p* < 0.05) in Biosynthesis of unsaturated fatty acids, Choline metabolism in cancer, and Glycerolipid metabolism and other pathways (Figures 6F,G). Through metabolic network analysis, Glycerophospholipid metabolism, Biosynthesis of unsaturated fatty acids, Sphingolipid signaling pathway, and Phospholipase D were found signaling pathways are the core pathways of differential metabolites (Figure 6H).

### 3.7 Correlation analysis between serum metabolism and IMF, TG

**Figure 7:**
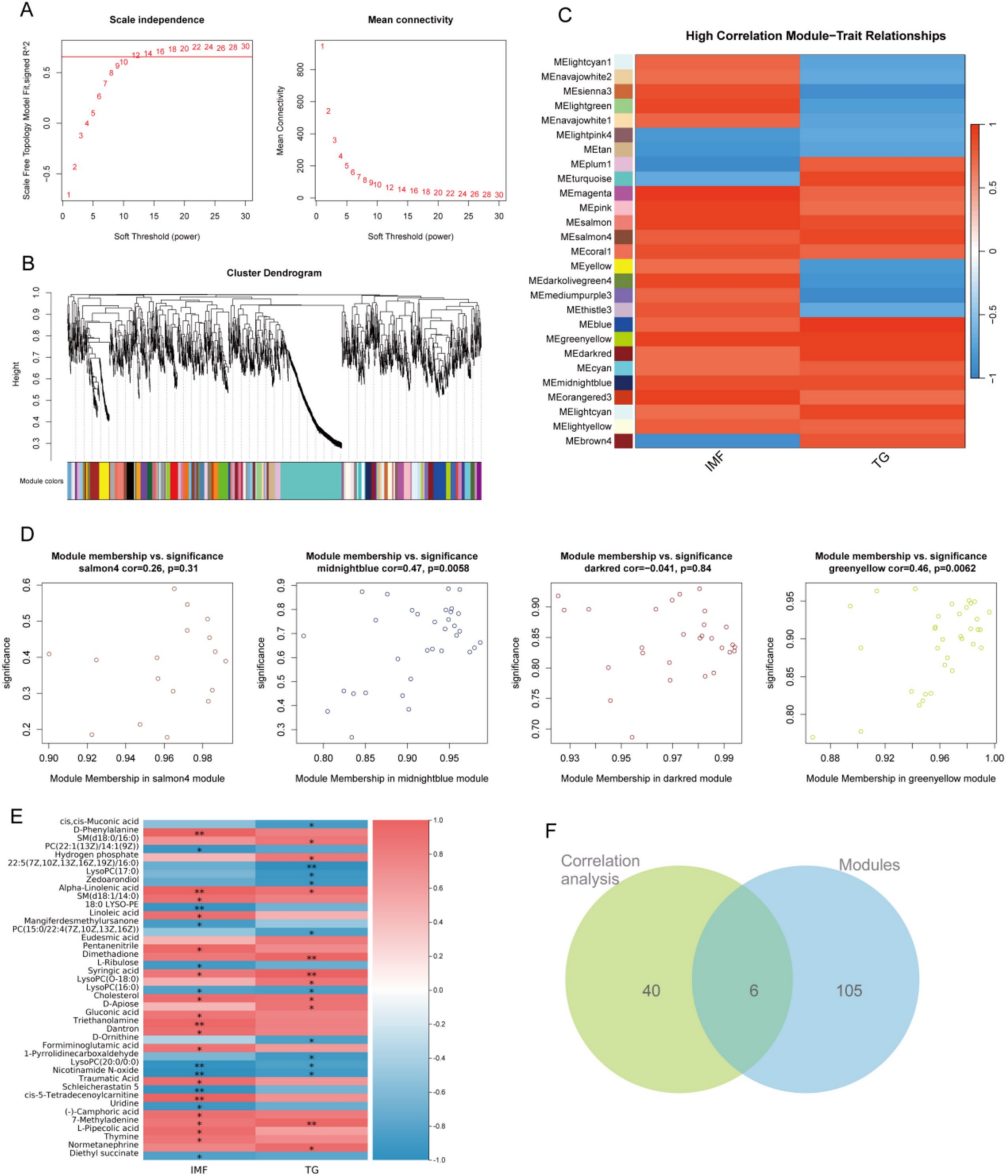
Weighted gene co-expression network analysis (WGCNA). **(A)** Scale independence (left) and mean connectivity (right) analysis for choosing the soft threshold in establishing the WGCNA network; **(B)** Gene clustering and identification of gene modules using WGCNA; **(C)** Heatmap of the relationship between gene modules and lipid traits. The relationships were assessed by calculating the Pearson correlation coefficients between the module eigengenes and lipid traits (IMF and TG); **(D)** Four core modules that are highly correlated with lipid traits; **(E)** Heatmap of the relationship between serum metabolites and lipid traits; **(F)** Venn of modules and association analysis.

In order to further ensure the accuracy of analysis and screen out the core metabolites that may affect lipid traits, we simulated the weighted gene correlation network analysis (WGCNA) algorithm commonly used in transcriptome analysis and tried to apply it to metabolomics analysis. The core metabolites were expected to be screened by modular classification of metabolism and association analysis by extracting module eigenvalues and lipid traits. In this study, the soft threshold was set as 12 (Figure 7A), and serum metabolites were divided into 89 modules according to their abundance characteristics (Figure 7B). The correlation between the eigenvalues of each module and lipid traits was analyzed. A total of 27 modules with strong correlation (R > 0.7) were screened out (Figure 7C). Four modules with the highest correlation were screened out and 105 serum metabolites were extracted from them (Figure 7D). Subsequently, correlation analysis between all serum metabolites and lipid traits was performed, and 40 serum metabolites with significant associations with lipid traits were obtained (Figure 7E). The results of WGCNA analysis and the results of correlation analysis were intersected, six potential core serum metabolites SM (d18:0/16:0), Hydrogen phosphate, Zedoarondiol, Syringic acid, 7-methyladenine, and Normetanephrine that might affect lipid traits were obtained (Figure 7F).

## 4 Discussion

Isobutyric acid is used as an organic acidifier in piglets’ diet in the present study. Isobutyric acid, also known as 2-methylpropionic acid, is one of the main energy providers for intestinal epithelial cells (27). Adding isobutyric acid to chicken diets can promote intestinal integrity and alter the composition of cecal microbiota (28). We found isobutyric acid could improve serum antioxidant ability and immunity, and promote intestinal mucosal barrier function, which may be related to the change of the composition of the cecal microbiota. Feeding gluconic acid influences the composition and activity of the intestinal microflora and may improve growth performance of piglets after weaning (29). Interestingly, we also found isobutyric acid increased IMF and muscular TG in LT of weaned piglets. Therefore, the supplementation with isobutyric acid may enhance intestinal mucosal barrier function and further improve meat quality.

Weaning stress is a major contributor to oxidative stress in blood of piglets. Malondialdehyde is the second reaction product of lipid peroxidation. MDA content can be used as the main product to measure the degree of oxidative stress (30). The activities of SOD and GST reflect the antioxidant status of the body (31). Isobutyric acid strengthened the stress resistance of weaned piglets, obviously increased T-AOC level and SOD activity (*p* < 0.01). For further verification of the effect of the isobutyric acid on oxidative stress, we analyzed the level of GST and MDA. In the current study, a diet with 0.5% isobutyric acid showed a significant decrease (*p* < 0.01) in the activity of GST and content of serum MDA. Previous studies showed that treatment with acetate and butyrate could reverse the production of MDA but increase levels of SOD in glomerular mesangial cells (32). Oral Epigallocatechin-3-gallate increase the production of propionate and butyrate, with an increases of T-AOC and SOD in mice (33). Therefore, it is necessary to investigate the changes of serum metabolites between IB group and CON group.

Immunoglobulins have antibody activity and play an important role in defending disease. We found Ig A significantly increased (*p* < 0.05) in IB group. Ig A can prevent pathogens from adhering to and invading mucosal epithelial cells, reducing the risk of infection (34). The result also showed an upregulated (*p* < 0.01) in Ig M. As the primary antibody of the initial immune response, Ig M is found mostly in the blood, which can activate the complement system and facilitate pathogen clearance (35). Therefore, feeding a diet supplemented with isobutyric acid can improve immunity in weaned piglets.

From birth to post-weaning, the piglets’ intestine undergoes radial changes from crypt stem cells to the villous surface and changes in transport function from the duodenum to the distal colon (36, 37). The mucus, villi, and tight junctions in the intestinal lumen together form the first barrier controlling the movement of materials in and out of the intestine (33). *MUC2* released by goblet cells is an important component of the mucus layer in the intestinal lumen (38). Tight junctions are located below the bottom of the microvilli. *ZO-1*, *Claudin-1* and *Occludin* form a histone scaffold together, which is important component in maintaining the integrity of the intestinal mucosal barrier (39). We further confirmed that isobutyric acid increased the expression of *Claudin-1* and *Occludin* in jejunum and ileum. Overall, these data demonstrated that isobutyric acid has a positive effect on intestinal morphology and mucosal barrier function in weaned piglets.

Microorganisms play an essential role in maintaining intestinal homeostasis (40). Probiotics and pathogenic bacteria in the intestinal tract constrain and regulate each other with the intestinal environment to uphold mucosal integrity and barrier function (41). Changes in diet can alter the composition of the intestinal flora, leading to changes in bacterial metabolites and the phenotype of the animal (42). In this study, we examined the composition of the gut microbiota. Our research results confirmed that the addition of isobutyric acid in the diet affects the composition and abundance of gut microbiota, leading to partial changes in the intestinal microbiota structure of weaned piglets. Firmicutes and Bacteroidetes, which together account for more than 90% of the total bacterial abundance in the cecum microbiota, are two major phyla. After the addition of isobutyric acid to the diet, the relative abundance of Firmicutes decreased, while Bacteroidetes and Verrucomicrobia increased. At the genus level, most bacteria belong to the phylum Firmicutes and produce short-chain fatty acids, especially acetate and butyrate (43). Acetate can induce immune responses to resist harmful bacteria, and when potentially harmful bacteria are present, acetate can trigger the production of Ig A in the colon, which binds to bacteria and inhibits their colonization and invasion in the mucosal layer (44). Butyrate is the preferred energy source for intestinal epithelial cells, and 75% of the energy of the intestinal mucosa comes from its metabolism through *β*-oxidation of hydroxybutyryl-CoA (45).

In order to further substantiate the potential link between intestinal morphology and cecal microbiota, we conducted a correlation analysis. The results showed that [Eubacterium]_coprostanoligenes, T34, Family_XIII_AD3011, Prevotellaceae_NK3B31, Prevotella, Solobacterium, UCG-005 and Parabacteroides were significantly positively correlated (*p* < 0.05) with VCR in small intestinal segments. Bai et al. observed that AUF1 stabilizes *MUC2* mRNA in goblet cells, and the increase of [Eubacterium]_coprostanoligenes_group induced *MUC2* mRNA depends on AUF1 expression (46).

Meanwhile, the correlation analysis between lipid traits and cecal microbiota showed Solobacterium and Erysipelotrichaceae were significantly positively correlated (*p* < 0.05) with IMF, and [Eubacterium]_coprostanoligenes, T34, Family_XIII_AD3011, Prevotellaceae_NK3B31, Prevotella, UCG-005 and Parabacteroides were significantly positively correlated with muscle TG. We found that these strains shared commonalities in their products. Most of their metabolites are short-chain fatty acids and short-chain fatty acid salts, especially butyric acid and butyrate, which are consistent with the changes in the intestinal flora structure (47). [Eubacterium]_coprostanoligenes_group, a cholesterol-reducing anaerobe in feces, has been found decreased in the HFD-induced hyperlipidemic rats and could generate beneficial SCFAs and have beneficial effects on dyslipidemia (48, 49). Previous study had shown that [Eubacterium]_coprostanoligenes_group can decompose cholesterol into sterols that cannot be absorbed in the intestine and is finally excreted with feces (50). In addition, [Eubacterium]_coprostanoligenes_group is considered to be the pivotal genus in the fecal microecosystem mediating the effect of HFD on dyslipidemia through sphingosine (51). This consistency may be one of the reasons why isobutyric acid enhances intestinal mucosal barrier function and promotes intramuscular fat deposition.

The dietary supplementation with 0.5% isobutyric acid increased the marbling score of muscle tissue of piglets. IMF is positively correlation with the meat quality, including the color, texture and flavor. As food, higher IMF substantially enhances the overall satisfaction of consumers (52). Our results showed that isobutyric acid elevated the IMF and TG in LT. Like other studies using short-chain fatty acid as a dietary additive, Huang et al. found that maternal butyrate supplementation lead to ectopic lipid accumulation in skeletal of offspring (18). Studies have found that the downregulation of adiponectin is interrelated with the tendency of pigs to accumulate fat, which is associated with our results (53). Adiponectin, an adipocyte-derived hormone, plays an important role in lipid metabolism and glucose homeostasis (54). In our study, isobutyric acid may regulate fat deposition through the *de novo* fatty acid synthesis (DNL) pathway. *ACC* and *FASN* are the key metabolic enzyme of the DNL pathway (55). We found that isobutyric acid significantly increased the expression of *ACC* and *FASN* in LT, which may be the cause of the IMF deposition we observed in the IB group. Moreover, isobutyric acid had a significantly inhibitory effect on *LPL*, *FABP4* and *HSL*., which are key genes involved in cellular fatty acid uptake and the rate-limiting gene for lipolysis. In addition, butyrate could enhance the formation of adipocytes by 10–20% and mRNA expression of adipocyte markers by 20–200% in procine SVF undergoing adipocyte differentiation (56).

Serum metabolomics could comprehensively analyze and identify the serum metabolites by using mass spectrometry. Untargeted serum metabolomics technologies have a wide range of applications in medicine, including the identification of disease markers, the analysis of pathological mechanisms and the screening of clinical animal models. However, fewer studies have used the serum metabolome to analyze serum markers associated with intramuscular fat in weaned piglets. In our study, isobutyric acid significantly altered (*p* < 0.05) the composition of serum metabolites in weaned piglets especially Lipids and lipid−like molecules, Organic acids and derivatives, and Organoheterocyclic compounds. KEGG showed a significantly enriched (*p* < 0.05) pathway related to lipid metabolism, for instance Biosynthesis of unsaturated fatty acids, Choline metabolism in cancer, and Glycerolipid metabolism and other pathways. And Glycerophospholipid metabolism is one of the core pathway of differential metabolites through metabolic network analysis. Glycerophospholipid metabolism is the process of biosynthesis, catabolism and transformation of intracellular glycerophospholipids. Glycerophospholipids are one of the main components of cell membranes and are involved in intracellular signaling, energy storage, and cellular recognition. Glycerophospholipid metabolism may be a potential regulator of the promotion of intramuscular fat deposition (30). Another core pathway is Phospholipase D signaling pathway. Phospholipase D hydrolyses phosphatidylcholine to produce phosphatidic acid and free choline. Phosphatidic acid can not only be converted to diacylglycerol by phosphatidic acid phosphohydrolase, but also deacylated by phospholipase A to produce lysophosphatidic acid (57). To summarize, these serum metabolites may affect the IMF content and meat quality by Glycerophospholipid metabolism and Phospholipase D signaling pathway.

The results of the correlation analysis showed that Alpha-Linolenic acid, Syringic acid, 7-methylidene and cholesterol are positively correlated with IMF and TG. Alpha-Linolenic acid is a polyunsaturated fatty acid, and animals cannot synthesize by themselves, must be taken in through dietary intake. Alpha-Linolenic acid inhibits inflammatory responses, boosts immunity and antioxidant ability (58). In the current study, isobutyric acid improve antioxidant capacities may be related to the upregulation of Alpha-Linolenic acid. Syringic acid is a phenolic compound that plays an important role in regulating fat metabolism and lowering the lipid content of the liver (49). Furthermore, we intersected the results derived from the WGCNA with the results of the correlation analysis, and we obtained six potential core serum metabolites. In addition to Syringic acid and Normetanephrine, we discovered four other related metabolites, such as SM (d18:0/16:0), Hydrogen phosphate, Zedoarondiol and 7-methylidene. Previous studies showed different doses of phosphate can regulate fat metabolism by affecting the expression of lipogenic and lipolysis genes (59). Sphingolipid metabolism may be associated with excess body fat (60). These results may explain the diet supplementation with isobutyric acid promote IMF deposition in weaned piglets.

In summary, the findings of our study demonstrate that diet supplementation with 0.5% isobutyric acid could promote intestinal mucosal barrier function and antioxidant ability, probably by changing the composition of cecal microbiota. Moreover, we speculated isobutyric acid may increase the deposition of IMF by regulating the composition of cecal microbiota, and subsequently regulating the serum metabolism to influence fat deposition in LT. Therefore, isobutyric acid may be a resultful addition in piglets diets to improve small intestinal mucosal barrier function. Meanwhile, isobutyric acid also promoted meat quality to improved nutritional value of pork in the window period.

## 5 Conclusion

**Figure 8:**
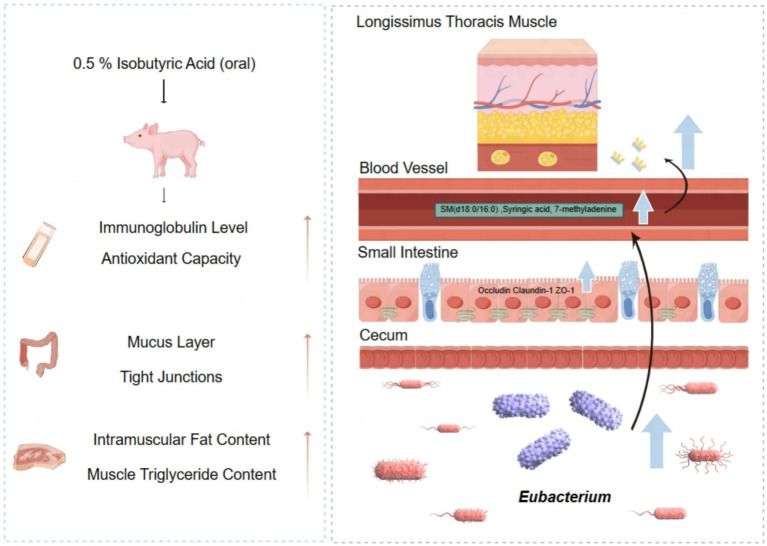
Effect of isobutyric acid on intestinal mucosal barrier function and meat quality of weaned piglets.

In summary, the findings of our study demonstrate that diet supplementation with 0.5% isobutyric acid could promote intestinal mucosal barrier function and antioxidant ability, probably by changing the composition of cecal microbiota. Moreover, we speculated isobutyric acid may increase the deposition of IMF by regulating the composition of cecal microbiota, and subsequently regulating the serum metabolis to influence fat deposition in LT (Figure 8). Therefore, isobutyric acid may be a resultful addition in piglets diets to improve small intestinal mucosal barrier function. Meanwhile, isobutyric acid also promoted meat quality to improved nutritional value of pork in the window period. However, it is essential to acknowledge that our studies may not reflect long-term effects, necessitating further chronic studies.

## Data Availability

The original contributions presented in the study are included in the article/Supplementary material, further inquiries can be directed to the corresponding author.

## Supplementary material

SUPPLEMENTARY FIGURE S1 Diversity analysis in two groups. **(A)** Rankabundance. **(B)** Rarefaction curves. **(C)** Alpha diversity analysis.

SUPPLEMENTARY TABLE S1 Primers used for gene expression analysis via RT-qPCR.

SUPPLEMENTARY TABLE S2 Composition and nutrient levels of the basal diet.

SUPPLEMENTARY TABLE S3 Effect of isobutyric acid on growth performance of weaned piglets.
