# Supplementary material for: Dietary isobutyric acid supplementation improves intestinal mucosal barrier function and meat quality by regulating cecal microbiota and serum metabolites in weaned piglets
Source: Front Vet Sci. 2025 May 2;12:1565216. doi: 10.3389/fvets.2025.1565216 (PMC12081365; doi:10.3389/fvets.2025.1565216)
Supplement: Supplementary file 1 [file Table_1.docx]

Supplementary Material

# Supplementary Figures and Tables

## Supplementary Table

**Table S1.** Primers used for gene expression analysis via RT-qPCR

| Primer name | Sequence (5'-3') |
| --- | --- |
| *β-actin-F* | ATGGTGAAGGTCGGAGTGAAC |
| *β-actin-R* | CTCGCTCCTGGAAGATGGT |
| *Claudin-1-F* | GATTTACTCCTACGCTGGTGAC |
| *Claudin-1-R* | CACAAAGATGGCTATTAGTCCC |
| *Occludin-F* | CTACTCGTCCAACGGGAAAG |
| *Occludin-R* | ACGCCTCCAAGTTACCACTG |
| *ZO-1-F* | CAGCCCCCGTACATGGAGA |
| *ZO-1-R* | GCGCAGACGGTGTTCATAGTT |
| *MUC2-F* | CTGCTCCGGGTCCTGTGGGA |
| *MUC2-R* | CCCGCTGGCTGGTGCGATAC |
| *ACC-F* | GGCCATCAAGGACTTCAACC |
| *ACC-R* | ACGATGTAAGCGCCGAACTT |
| *FASN-F* | GTCCTGCTGAAGCCTAACTC |
| *FASN-R* | TCCTTGGAACCGTCTGTG |
| *FATP1-F* | GGACCTCTGTCTTAGCCTCACT |
| *FATP1-R* | CGCATCCTTCAGTCTTTGTTCT |
| *FABP4-F* | CTGAGATTGCCTTCAAATTG |
| *FABP4-R* | CTTGGCTTATGCTCTCTCATA |
| *LPL-F* | CTCGTGCTCAGATGCCCTAC |
| *LPL-R* | GGCAGGGTGAAAGGGATGTT |
| *HSL-F* | CTTTCGCACCAGCCACAAC |
| *HSL-R* | CTCGTCGCCCTCAAAGAAGA |
| *PPARα-F* | AGAGCCCCATCTGTCCTCTC |
| *PPARα-R* | ACTGGTAGTCTGCAAAACCAAA |
| *SREBP1c-F* | AGCGGACGGCTCACAATG |
| *SREBP1c-R* | CGCAAGACGGCGGATTTA |
| *DGAT1-F* | TGGACTACTCACGCATCAT |
| *DGAT1-R* | GTGGAAGAGCCAGTAGAAGAA |

*ZO-1*, zonula occludens-1; *MUC2*, mucin-2; *ACC*, acetyl-CoA carboxylase; *FASN*, fatty acid synthase; *FATP1*, fatty acid transport protein 1; *FABP4*, fatty acid-binding protein 4; *LPL*, lipoprotein lipase; HSL, hormone-sensitive lipase; *PPARα*, peroxisome proliferator-activated receptor α; *SREBP1c*, sterol regulatory element binding protein 1c; *DGAT1*, diacylglycerol-O-acyltransferase 1.

**Table S2.** Composition and nutrient levels of the basal diet.

| Ingredients | Content (%) | Calculated nutrient levels |  |
| --- | --- | --- | --- |
| Corn | 54.7 | Digestible energy, MJ·kg^-1^ | 14.21 |
| Soybean meal | 17 | Crude protein, % | 20.49 |
| Extruded soybean | 8 | Lysine, % | 1.27 |
| Fish meal | 5 | Methionine + Cystine, % | 1.63 |
| Whey powder | 10 | Threonine, % | 0.93 |
| Soybean oil | 1 | Isoleucine, % | 0.82 |
| Dicalcium phosphate | 0.4 | Calcium, % | 0.73 |
| Calcium carbonat | 0.7 | Total phosphorus, % | 0.65 |
| L-Lysine-HCl | 0.15 | Available phosphorus, % | 0.38 |
| L-Threonine | 0.13 |  |  |
| DL-Methionine | 0.92 |  |  |
| L-Tryptophan | 0.7 |  |  |
| Salt | 0.3 |  |  |
| Premix^1^ | 1 |  |  |
| Total | 100 |  |  |

^1^ The premix provided the following per kilogram of diets：vitamin A 10,500 IU, vitamin D_3_ 400 IU, vitamin E 15 mg, vitamin K_3_ 3 mg, vitamin B_1_ 2 mg, vitamin B_2_ 12 mg, vitamin B_6_ 6 mg, vitamin B_12_ 0.05 mg, biotin 0.2 mg, folic acid 2 mg, D-calcium 25 mg, Fe 100 mg, Cu 80 mg, Mn 50 mg, Zn 90 mg, Co 1 mg, Se 0.3 mg, I 0.15 mg.

**Table S3.** Effect of isobutyric acid on growth performance of weaned piglets.

| Items | CON^1^ | IB^1^ | SEM^2^ | P-value |
| --- | --- | --- | --- | --- |
| Initial weight, kg | 9.29±0.74 | 9.72±0.91 | 0.31 | 0.563 |
| Final weight, kg | 16.98±1.02 | 17.90±0.43 | 0.35 | 0.229 |
| ADG, g/d | 366.33±36.96 | 373.33±28.29 | 12.12 | 0.807 |

^1^CON, control group; IB, isobutyric acid group; ^2^SEM,standard error of the mean. ADG, average daily gain. Results are presented as mean ± SD, n = 3. * p < 0.05 and ** p < 0.01

## Supplementary Figures


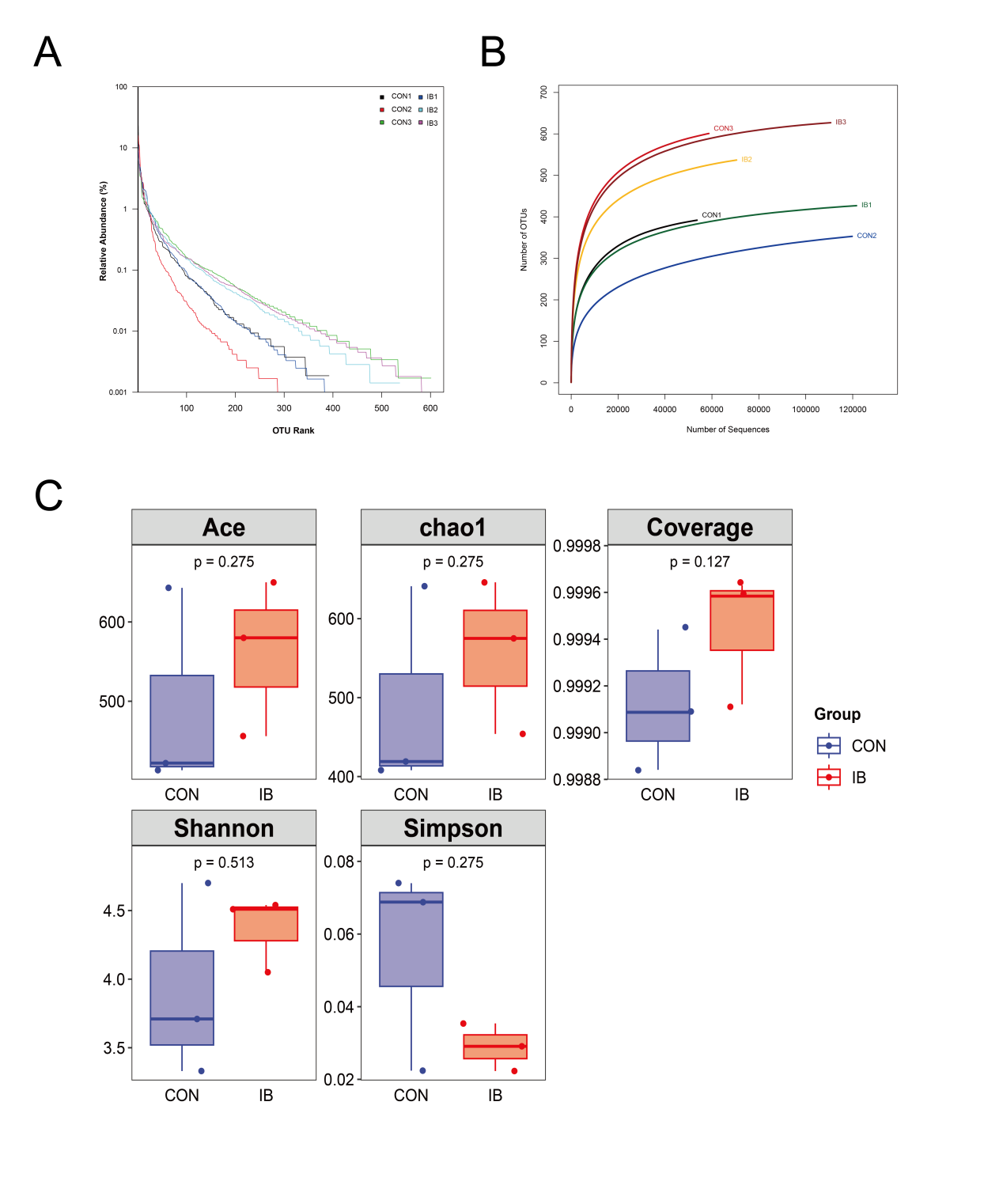


**Supplementary Figure S1.** Diversity analysis in two groups. (A) Rankabundance. (B)Rarefaction curves. (C) Alpha diversity analysis.
